# Teliosporogenesis of the Peanut Smut Fungus *Thecaphora frezzii* in *Arachis hypogaea*: A Correlative Multiscale Microscopy Study

**DOI:** 10.3390/plants15050841

**Published:** 2026-03-09

**Authors:** María Florencia Romero, Orlando F. Popoff, Guillermo J. Seijo, Ana Maria Gonzalez

**Affiliations:** 1 Universidad Nacional del Nordeste, Instituto de Botánica del Nordeste (UNNE-CONICET), Facultad de Ciencias Agrarias, Corrientes CP 3400, Argentina; mariafloromero@gmail.com; 2 Universidad Nacional del Nordeste, Instituto de Botánica del Nordeste (UNNE-CONICET), Facultad de Ciencias Exactas y Naturales y Agrimensura, Corrientes CP 3400, Argentina; popoff@exa.unne.edu.ar (O.F.P.); jgseijo@exa.unne.edu.ar (G.J.S.)

**Keywords:** confocal laser scanning microscopy, correlative microscopy, fungal development, histology, peanut smut, plant–fungus interaction, smut fungi, teliosporogenesis, teliospore balls, *Thecaphora frezzii*

## Abstract

The smut fungus *Thecaphora frezzii* causes severe yield losses in peanuts (*Arachis hypogaea*) in Argentina. Previous work established its fully intracellular biotrophic progression through subterranean organs and its exclusive sporulation within the seed coat, yet the ontogeny of teliospore formation in planta remained unresolved. Here, we applied a pragmatic correlative multiscale microscopy approach based on serial paraffin sections examined by stereomicroscopy, light microscopy, confocal laser scanning microscopy, and scanning electron microscopy, enabling spatial correlation of fungal structures within their tissue context. Using this integrative framework, we characterized the organization and progression of sporogenic structures associated with teliosporogenesis. Teliosporogenesis proved to be tightly synchronized with host tissue context and seed developmental stage, and was consistently preceded by a marked reorganization of sporogenous hyphae into three-dimensional coiled hyphal aggregates embedded in a mucilaginous matrix. These precursors undergo hyphal fragmentation followed by central–peripheral differentiation, whereby a small number of central units enlarge and individualize into teliospore initials while peripheral elements collapse, yielding stable teliospore balls as the final sporogenic product. This developmental sequence defines a distinct ontogenetic pattern not captured by current schemes of sporogenesis, here designated the Teliospore-ball type. Our results clarify the developmental pathways of *T. frezzii* sporulation in planta and demonstrate how accessible multiscale microscopy can be used to integrate structural information across spatial scales in complex plant–fungus interactions.

## 1. Introduction

Peanut smut, caused by the basidiomycete *Thecaphora frezzii* Carranza & Lindquist, is currently one of the most destructive diseases affecting *Arachis hypogaea* L. production in Argentina [1]. Since its first detection in Córdoba (Argentina) in 1995, this soilborne disease has rapidly spread throughout the peanut-growing region, reaching pod incidences of 50–70% in highly infested fields and causing yield losses of up to 35% [2,3]. The disease is characterized by the replacement of kernels with compact, dark, teliospore masses that remain hidden until pods are unearthed [2]. The long-term persistence of teliospores in soil [4], the inconsistent performance of fungicides [5], and the limited availability of resistant cultivars [6,7,8,9] collectively render *T. frezzii* a major threat to the sustainability of peanut cultivation in Argentina [10].

A series of studies conducted over the last decade have partially improved our understanding of the fungal life cycle, infection biology, and early colonization of peanut plants by *T. frezzii* [1,11,12,13,14]. More recently, our previous contribution on this pathosystem provided extensive experimental evidence on the colonizing pathways of the fungi through peanut tissues until sporulation. We demonstrated that the pathogen enters via the gynophore (peg) during early subterranean development (R2–R3 stages), then invades the young pericarp, funiculus, and ultimately the seed coat. Microstructural analyses further reveal that the fungus follows vascular bundles intracellularly, inducing hypertrophy, hyperplasia, and progressive disorganization of the host mesocarp and seed coat. These anatomical disruptions coincide with the onset of teliospore formation in seed coat, which ultimately arrests embryo development and replaces the kernel tissue when the infection occurs early during embryogenesis [15]. Despite these detailed histopatological descriptions, the ontogeny of spore balls during in vivo sporulation remained less understood, and the cellular events that drive teliosporogenesis in *T. frezzii* could not be addressed in detail so far.

Teliosporogenesis is a defining feature of smut fungi and has long been used as a taxonomic and phylogenetic criterion within the Ustilaginomycetes [16,17,18,19,20,21]. Classical studies using light and transmission electron microscopy describe four major ontogenetic patterns—*Ustilago* (Pers.) Roussel, *Microbotryum* Lév., *Tilletia* Tul. & C. Tul., and *Entorrhiza* C.A. Weber, types—distinguished by how sporogenous hyphae segment, how teliospore initials are enveloped, and how the spore wall layers (sheath, exosporium, middle layer, endosporium) are deposited [16,17]. However, all these types were proposed for smuts that produce single mature spores.

In spore-ball-forming genera such as *Sorosporium* F. Rudolphi, despite teliospores typically originating from coiled sporogenous hyphae embedded in a hyaline matrix [22], they have traditionally been assigned to the *Ustilago* type. However, considerable variability exists in ball cohesion, wall structure, and germination patterns among genera forming spore balls [21,23]. Moreover, *Thecaphora* as currently circumscribed is morphologically heterogeneous, and many species—including those infecting dicot hosts—exhibit developmental pathways not easily assignable to the classical ontogenetic types [16,19,23]. For *T. frezzii* in particular, the developmental mechanism by which permanent teliospore balls originate and mature within the seed coat has not yet been clearly linked to any previously described ontogenetic pattern.

Although teliosporogenesis has been documented in multiple smut pathogens—including *Ustilago maydis* (DC.) Corda, *Ustilago scitaminea* Syd., *Thecaphora deformans* Durieu & Mont. and *Tilletia* spp.—most developmental models are derived from in vitro systems or from fragmented in planta observations that do not allow precise correlation between fungal stages and host tissue context [16,19,24,25]. Correlative microscopy workflows that integrate light/fluorescence imaging with electron microscopy can resolve developmental processes across scales, yet their use in plant pathology remains limited by organ size, anatomical complexity, and difficulties in achieving spatial correspondence across modalities [26,27,28,29,30]. These challenges are exacerbated in the peanut–*T. frezzii* system, where the pathogen develops asynchronously across large subterranean reproductive structures, making it difficult to reconstruct teliosporogenesis in situ [15].

Here, we adapted a pragmatic correlative workflow based on conventional histological tools to enable multimodal and multiscale imaging of adjacent serial sections from the same tissue block. By combining stereomicroscopy, histological staining, fluorescence imaging, and scanning electron microscopy on near-identical regions of interest, we achieved spatially resolved analysis of fungal development during in planta teliosporogenesis in the seed coat. Specifically, we aimed to: (i) document the differentiation of sporogenous hyphae and the formation of spore ball initials; (ii) reconstruct the ontogenetic progression from early precursor structures to mature teliospore-ball organization; (iii) characterize the host anatomical and cytological changes associated with sporulation; and (iv) propose an ontogenetic model for teliospore-ball formation in *T. frezzii* within a comparative framework of Ustilaginomycetes.

## 2. Materials and Methods

### 2.1. Plant Material

Peanut plants (*Arachis hypogaea*) of the commercial runner type cv. Granoleico were obtained from El Carmen Nursery (General Cabrera, Córdoba, Argentina; 32°49′40.8″ S, 63°52′14.0″ W). This highly susceptible material was selected from a smut resistance evaluation trial including different landraces and breeding lines performed during the 2022–23 and 2023–24 growing seasons. The soil at the site was inoculated to reach approximately 1.2 × 10^4^ teliospores g^−1^. These levels were approximately 3.5 times higher than the maximum inoculum density reported in commercial production fields. The material used here as susceptible was evaluated during two consecutive growing seasons using a randomized complete block design with three replications per genotype [6,7]. Briefly, each experimental unit consisted of 25 plants grown in 2.5 m rows. Disease incidence and severity assessment was performed at harvest. For doing that, 100 pods per plot were randomly sampled and manually opened. For each pod, the number of infected seeds and the degree of infection were recorded. Standard agronomic practices were applied throughout the cycle [6,7].

Reproductive structures, including pegs and pods from developmental stages R4 to R9 as defined by Boote [31], were collected. R4 (full pod) corresponds to a fully expanded pod, without significant seed development; R5 (beginning seed) and R6 (full seed) represent progressive cotyledon growth and complete occupation of the pod cavity; R7 (beginning maturity) is characterized by the appearance of natural coloration or visible blotches on the internal pericarp or seed coat; R8 (harvest maturity) shows a yellowish-brown to dark brown pericarp; and R9 (over-mature pod) presents orange or light brown coloration associated with natural tissue deterioration. Because peanuts are an indeterminate growth species, all stages (R4–R9) were collected simultaneously from the same experimental plots almost at harvest time (130–140 days).

The samples were fixed in FAA (formaldehyde: ethanol: acetic acid, 5:90:5) directly in the field, and stored in FAA or alcohol 70° for histological processing [32]. For each reproductive stage (from R4 to R9), five symptomatic fruits were selected from ten representative plants. A total of 30 fruits were processed, from which 30 paraffin blocks were prepared. Serial sections were obtained from each block. For microscopic analysis, the number of slides was variable, due to size differences among samples, small fruits allowed up to approximately 10 serial sections to be mounted on a single slide, whereas large fruits (whole pods or large ROIs) typically allowed only one or two sections per slide.

### 2.2. Correlative and Multiscale Microscopy Workflow

To achieve a multimodal and spatially resolved analysis of fungal colonization and teliospore development in peanut tissues, we implemented a correlative microscopy workflow that integrates stereomicroscopy (SM), light microscopy (LM), confocal laser scanning microscopy (CLSM), and scanning electron microscopy (SEM). This approach is based on the use of a single paraffin-embedded tissue block from which serial sections are mounted onto at least four glass slides, enabling the comparative observation of identical tissue regions with complementary techniques. The detailed procedures for each technique are described in the following subsections.

#### 2.2.1. Stereomicroscopy

Young and mature fruits and seeds previously fixed in FAA were carefully dissected under a Leica DM LV2 stereomicroscope (Leica Microsystems GmbH, Wetzlar, Germany) by cutting them in half using a single-edge blade and immediately photographed. Observations and high-resolution photographic documentation were performed using the same stereomicroscope equipped with a Leica Flexacam camera (Leica Microsystems GmbH, Germany) prior to tissue embedding.

#### 2.2.2. Embedding and Serial Sectioning

Based on stereomicroscopic observations, representative regions of interest (ROIs) were dehydrated in an ascending ethanol series, pre-infiltrated with tertiary butanol, and embedded in paraffin following standard procedures [32,33]. Paraffin blocks were sectioned using a MICROM HM325 rotary microtome to obtain serial transverse and longitudinal sections of 10–12 μm thickness. Either individual sections (for large ROIs) or ribbons with multiple sections (for small fruits or ROIs) were mounted on separate microscope slides. In the case of large samples such as whole fruits (up to 2–3 cm long and ~1 cm wide), only one section could be mounted per slide, whereas smaller materials/ROIs allowed multiple sections per slide. This set of slides were used at LM, CLSM, and SEM analyses.

The slide destined for SEM was trimmed to approximately 4 cm in length prior to mounting the paraffin ribbon, to ensure it would fit in the chamber of the critical point dryer. Sections were affixed to the slides using Haupt’s adhesive [34] and allowed to dry for 48 h before further processing. This strategy ensured optimal adherence and preservation of tissue integrity during the various subsequent microscopy procedures.

#### 2.2.3. Light Microscopy (LM)

The sections were deparaffinized and stained using the Safranin–Astra Blue protocol [35]. In this double-staining protocol, safranin stains lignified cell walls and nuclei in red, whereas Astra Blue selectively stains cellulose-rich tissues in blue. In addition, safranin consistently stains the fungal structures of *T. frezzii*, including hyphae and teliospores, allowing their clear visualization within host tissues [36]. Stained sections were dehydrated, cleared, and permanently mounted in synthetic Canada balsam [37]. High-magnification observations and imaging were performed using a Leica DM LB2 light microscope equipped with a Leica ICC50 HD digital camera (Leica Microsystems GmbH, Germany). Large histological sections mounted in synthetic Canada balsam were also examined under a stereomicroscope equipped with transmitted light to document overall tissue context and to guide the selection of regions for subsequent high-magnification LM, CLSM, and SEM analyses.

#### 2.2.4. Confocal Laser Scanning Microscopy (CLSM)

Confocal images were acquired using a Stellaris 8 White Light Laser inverted confocal microscope (Leica Microsystems, Wetzlar, Germany) equipped with HC PL APO 10× dry (NA 0.4) and 63× oil immersion (NA 1.4) objectives. Serial optical sections were acquired in xyz mode to generate z-stacks for volumetric visualization. Single-channel images were combined into RGB composites using Leica LAS X Stellaris Compass software, version 5.3.0, assigning blue, green, and red pseudocolors to their respective spectral detection windows. Three-dimensional (3D) reconstructions were generated from stacked optical sections to visualize the spatial organization and continuity of fungal structures within host tissues.

Serial sections from a single paraffin block destined for CLSM comprised two main preparation protocols:

1. Safranin–Astra Blue-stained sections. Sections previously stained for LM and permanently mounted in synthetic Canada balsam were directly examined under CLSM.
2. Deparaffinized and rehydrated sections. Sections were deparaffinized and sequentially rehydrated to distilled water prior to fluorescence examination.

The following fluorescence detection procedures were applied as appropriate:

- Autofluorescence detection: Both previous groups were analyzed. To analyze native fluorescence from fungal and host cell walls, rehydrated sections were mounted in pure glycerol and observed without staining (protocol 1), taking advantage of the intrinsic autofluorescent properties of plant and fungal cell walls widely used in confocal studies [38,39,40]. Images were acquired using three spectral detection windows corresponding to blue (413–489 nm), green (508–633 nm), and red (635–750 nm) emission ranges. Excitation wavelengths were selected at 405 nm, 488 nm, and 638 nm from the White Light Laser spectrum. To minimize spectral cross-talk and bleed-through, channels were acquired in sequential mode (frame-by-frame) using identical scan settings for all channels within each region of interest. Safranin–Astra Blue-stained sections (protocol 2) were examined under the same spectral detection windows to detect fluorescence associated with lignified cell walls and fungal structures.
- Calcofluor White staining: Rehydrated sections (protocol 1) were stained with a mixture of one drop of 0.1% Calcofluor White M2R (Sigma-Aldrich, St. Louis, MI, USA)—which binds to 1,4-linked polymers such as cellulose and chitin—and one drop of 10% potassium hydroxide solution. The mixture was applied directly to the rehydrated section, covered with a coverslip, and observed immediately [41,42]. Calcofluor fluorescence was excited using a 405 nm wavelength selected from the White Light Laser spectrum, and emission was collected within the 413–489 nm detection window, according to the dye’s spectral properties [43].

#### 2.2.5. Scanning Electron Microscopy (SEM)

Slides previously prepared and trimmed for SEM were deparaffinized in xylene (two changes of 5 min each in pure xylene) and dehydrated through an ascending acetone series. Afterward, the slides were dried to the critical point using CO_2_ in a Denton Vacuum device (Denton Desk II, Moorestown, NJ USA) and mounted on aluminum stubs using double-sided carbon adhesive tape. Finally, they were sputter-coated with a 30–40 nm gold–palladium layer [44]. Observations and photomicrographs were carried out at 15 kV using JEOL 5800 LV (JEOL Ltd., Tokyo, Japan) and ZEISS EVO 15 (Carl Zeiss AG, Oberkochen, Germany) scanning electron microscopes at the Electron Microscopy Service of Universidad Nacional del Nordeste, Corrientes, Argentina.

## 3. Results

### 3.1. Correlative and Multiscale Microscopy Workflow in the Peanut Smut Pathosystem

The peanut smut pathosystem (*Arachis hypogaea*–*Thecaphora frezzii*) involves fungal colonization and teliospore formation across successive stages of pod and seed development. These processes span multiple spatial scales—from the whole fruit to individual fungal structures—and take place within host tissues undergoing marked ontogenetic changes. As a consequence, the fungal network develops complex three-dimensional architectures that cannot be fully understood using a single microscopy technique or a single focal plane.

To address this structural and developmental complexity, we implemented a correlative multiscale microscopy workflow integrating stereomicroscopy (SM), light microscopy (LM), confocal laser scanning microscopy (CLSM), and scanning electron microscopy (SEM). The workflow is based on serial sections obtained from the same paraffin-embedded tissue block, allowing spatial correspondence between imaging modalities at the level of tissues and biological structures. Rather than aiming to image the exact same microscopic field across techniques, this approach correlates defined biological targets across spatial scales, anatomical context, complementary contrast mechanisms, and increasing depth of field and resolution, in accordance with the principles outlined by Caplan et al. [27].

**Figure 1:**
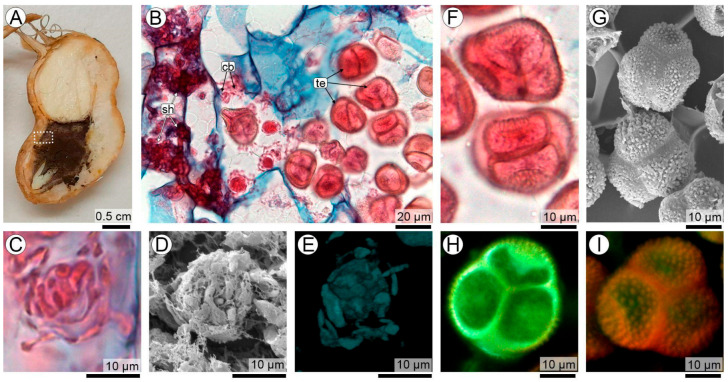
Correlative multiscale microscopy workflow applied to teliosporogenesis in the peanut smut pathosystem. (**A**) Stereomicroscopic view of an infected peanut seed showing the selected region of interest (ROI). (**B**) Light microscopy (LM) overview of the same ROI, which contains multiple stages of teliosporogenesis, including sporogenous hyphae (sh), coiled balls (cb), and teliospore balls (te) at different developmental stages. (**C**–**E**) Spore balls at early stages of teliosporogenesis visualized by (**C**) LM, (**D**) SEM on histological sections, and (**E**) CLSM 3D projection from stacked optical sections. (**F**–**I**) Fully developed, mature teliospore balls visualized by (**F**) LM, (**G**) SEM, and (**H**,**I**) CLSM 3D projections from stacked optical sections.

The correlative logic of the workflow is illustrated in Figure 1, which provides a visual guide to how complementary microscopy techniques are integrated in this study. A region of interest (ROI) is first identified at the organ level within an infected peanut seed (Figure 1A) and subsequently examined at the tissue level by LM (Figure 1B). This step establishes the anatomical location and developmental context in which fungal differentiation occurs and serves as the reference framework for subsequent analyses.

To exemplify the correlative strategy, two fungal structures representing different stages of teliosporogenesis are selected in Figure 1 and shown here solely to illustrate the application of correlative microscopy: *spore balls* and *teliospore balls*. Their morphology, ontogenetic significance, and developmental interpretation are addressed in detail in subsequent sections. In this figure, the same biological targets are examined across complementary imaging modalities to highlight the synergistic information gained from their integration.

In this context, the correlative multiscale approach was applied across multiple fungal structures involved in teliosporogenesis, including early spore-ball aggregates and fully mature teliospore balls as representative examples (Figure 1C–I). Light microscopy (LM) consistently resolved the histological framework and overall morphology of these structures within the seed coat, preserving their relationship with surrounding host tissues at both early and mature stages. Scanning electron microscopy (SEM), performed directly on corresponding histological sections, complemented LM by enhancing depth of field and revealing surface architecture and spatial organization, from the compact arrangement of early coiled aggregates to the ornamented surfaces and spatial relationships of adjacent teliospores in mature balls. Confocal laser scanning microscopy (CLSM) further extended this analysis by enabling optical sectioning and three-dimensional reconstruction, allowing internal organization, volumetric structure, and spatial continuity to be resolved in both developing and mature sporogenic structures. These examples illustrate how the integration of LM, SEM, and CLSM was applied throughout the study to analyze the multiple structural transitions involved in teliosporogenesis while preserving anatomical context and developmental continuity.

Overall, this workflow integrates (i) identification of specific biological targets, (ii) their placement within a broader anatomical context, (iii) complementary information derived from different contrast mechanisms and probes, and (iv) progressive increases in depth of field and spatial resolution. This integrated approach provides the technical framework for the multiscale analysis of fungal structures within host tissues, as applied in the following sections.

### 3.2. Seed Invasion and Early Colonization of the Seed Coat

**Figure 2:**
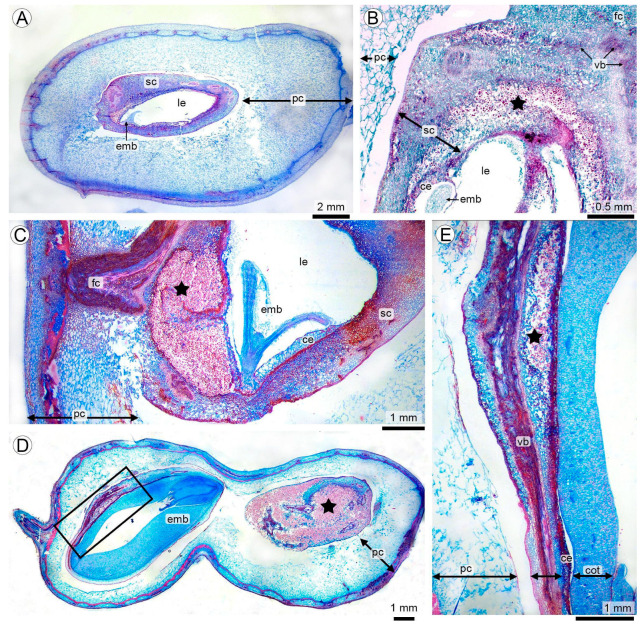
Seed colonization and teliosporogenesis by *T. frezzii* (**A**,**C**–**E**: Safranin–Astra Blue–stained histological sections observed under transmitted light stereomicroscopy, **B**: LM). (**A**) Early infection of pericarp with initial hyperplasia of the seed-coat parenchyma (R5 stage). (**B**) Detail of funiculus–seed region with marked hyperplasia of the parenchyma and early teliosporogenesis (★). (**C**) Teliosporogenesis of the hyperplastic seed coat in early infections (R6). (**D**) Comparison of late (left) versus early (right) infections within a single mature pod, showing embryo completion versus embryo arrest (R8). The boxed area indicates the hypertrophic seed-coat region. (**E**) Detail of the seed coat illustrating the transition from nearly normal tissue to hyperplastic, teliospore-filled regions (★), corresponding to the boxed area in (**D**). Abbreviations: ce: cellular endosperm; cot: cotyledon; emb: embryo; fc: funiculus; le: liquid endosperm; pc: pericarp; sc: seed coat; vb: vascular bundle.

In the subterranean fruits, *T. frezzii* reaches the seed through the funiculus after progressing along the peg into the pod. Hyphae advance preferentially along the vascular traces that connect the placenta with the funiculus and the seed tissues, and from these bundles they spread into the seed-coat parenchyma (Figure 2A,B). Once inside, they grow intracellularly through the parenchyma associated with the vascular strands and begin to proliferate actively within the seed coat (Figure 2B).

The colonized region displays a pronounced hyperplasia of the seed-coat parenchyma, together with an increase and branching of vascular bundles. Hyperplasia develops mainly in the inner parenchymatous layers of the testa, beneath an outer epidermis that remains structurally intact. This expanded region is consistently located near the funiculus–embryo axis, where young tissues are present (Figure 2B,C).

The developmental stage at the moment of colonization strongly determines the fate of the seed. When infection occurs at early embryogenic stages (R4–R6, torpedo embryo), embryogenesis arrests, and the hyperplastic seed coat ultimately replaces the entire kernel tissue content, becoming filled with teliospores (Figure 2D, right). Only sparse remnants of vascular strands persist by the end of the process. In contrast, when colonization begins later (R7–R8), the embryo completes development; although the seed coat still undergoes hyperplasia, cotyledons remain intact and structurally preserved (Figure 2D, left; Figure 2E).

### 3.3. Teliospore Development in the Seed Coat

**Figure 3:**
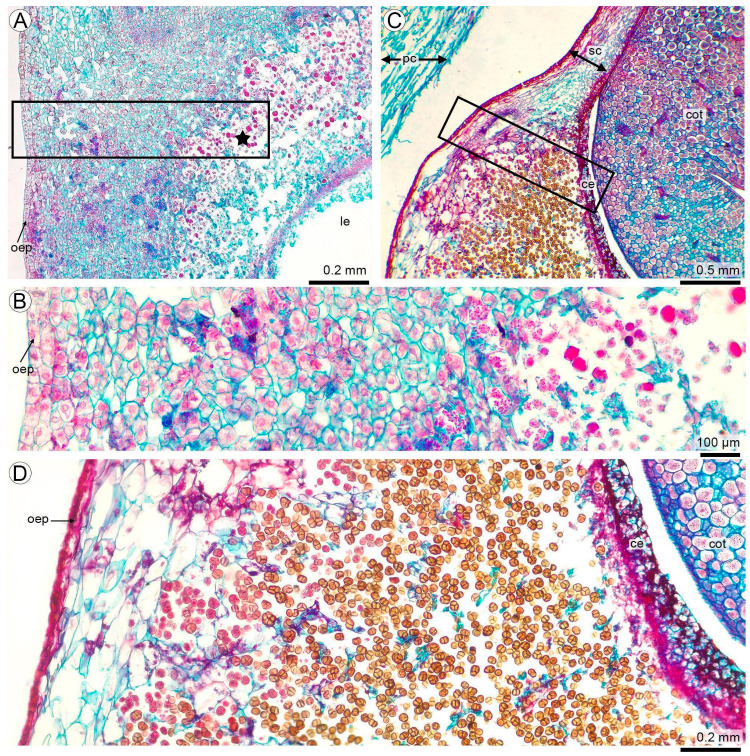
Teliosporogenesis of *T. frezzii* in the seed coat (LM). (**A**) Early teliospore formation in the hyperplastic seed-coat region near the funiculus–embryo axis (R6 stage); boxed area corresponds to (**B**). (**B**) Seed-coat parenchyma showing variable compaction and early stages of teliosporogenesis (★). (**C**) Inner-to-outer progression of teliosporogenesis in the seed coat (R8 stage); boxed area corresponds to (**D**). (**D**) Coexistence of young (left) and mature teliospores within hypertrophied, lysing host cells (right), illustrating the asynchronous and continuous nature of teliospore development. Abbreviations: ce: cellular endosperm; cot: cotyledon; le: liquid endosperm; oep: outer epidermis; pc: pericarp; sc: seed coat.

When colonization occurred at stages R6–R8, teliospore development took place within the hyperplastic seed-coat region surrounding the funiculus–embryo axis. Teliosporogenesis progressed centrifugally, beginning in the inner layers of the seed coat and extending toward the outer regions (Figure 3A). The hyperplastic seed coat consisted of parenchyma with highly variable degrees of compaction: cells appeared tightly packed beneath the outer epidermis and around vascular bundles, while inner regions displayed increasing intercellular spaces, producing a spongy texture (Figure 3B).

As the seed advanced from R6 to R8, parenchyma adjacent to the outer epidermis enlarged, and fungal proliferation intensified throughout the hyperplastic zone (Figure 3C). Teliosporogenesis was a continuous process, as indicated by the coexistence of early and advanced stages within adjacent seed-coat regions: young teliospores were observed within hypertrophied host cells undergoing lysis, while fully mature, darkly stained teliospores accumulated in nearby areas (Figure 3C,D).

### 3.4. Hyphal Colonization of the Endosperm and Embryo Region

**Figure 4:**
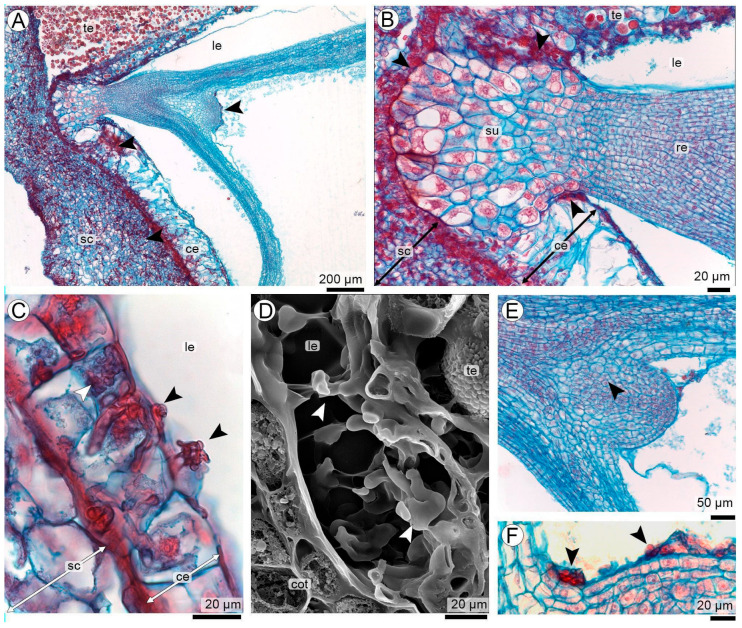
*Thecaphora frezzii* hyphal colonization of the cellular and liquid endosperm during early seed invasion (**A**–**C**,**E**,**F**: LM). (**A**) Torpedo-stage embryo surrounded by a peripheral band of cellular endosperm; hyphae from the seed coat reach the outer cellular layers. (**B**) Suspensor–radicle region with dense hyphae adjacent to the cellular endosperm, which shows slight cell enlargement but no hyperplasia; embryonic tissues remain unpenetrated. (**C**) Hyphae extending from the cellular endosperm toward the liquid endosperm. (**D**) SEM image showing hyphae within the liquid endosperm and spreading over the embryo surface without penetration. (**E**) Cellular endosperm near the suspensor with localized hyphal presence and minimal cellular enlargement. (**F**) Detail of hyphae spreading over the embryo surface, with no evidence of tissue penetration. Abbreviations: ce: cellular endosperm; cot: cotyledon; le: liquid endosperm; re: radicle; sc: seed coat; su: suspensor; te: teliospores, arrowheads indicate hyphae.

At R5 stage, the embryo had reached the torpedo stage, with a well-defined suspensor–radicle region and a surrounding peripheral band of cellularized endosperm approximately 3–5 layers thick (Figure 4A). Beyond the peripheral cellular layers, the embryo sac was filled by a large vacuole-like liquid endosperm (Figure 4A,B).

Hyphae advancing from the hyperplastic seed coat reached this cellular endosperm, where they extended between or along the outermost cellularized layers. Although hyphae densely colonized the cellular endosperm, this tissue showed no true hyperplasia; only a slight enlargement of cells was observed near the suspensor, without evidence of cell divisions, in sharp contrast with the pronounced hyperplasia of the seed coat (Figure 4B).

Occasional hyphal projections extended from the cellular endosperm into this liquid compartment (Figure 4C,D). A few of these hyphae grew sparsely along the surface of the developing embryo, without penetrating the embryo or the suspensor at this stage (Figure 4D–F).

### 3.5. Infective and Sporogenous Hyphae in the Seed Coat

**Figure 5:**
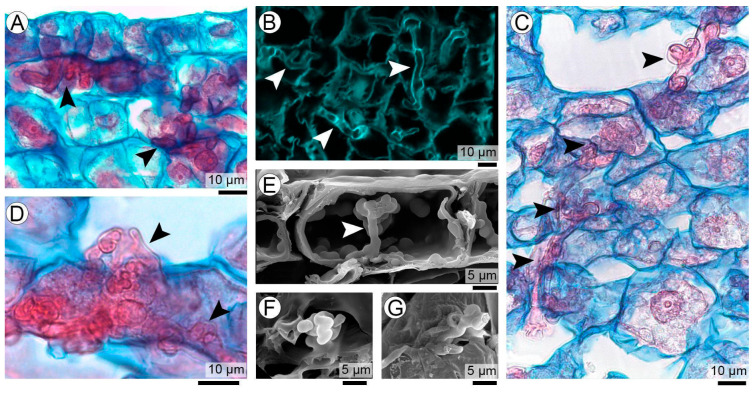
Progression and spatial pattern of infective and sporogenous hyphae of *T. frezzii* during peanut seed-coat colonization. (**A**,**C**,**D**: LM). (**A**) Infective hyphae growing intracellularly within host parenchyma cells. (**B**) CLSM image with Calcofluor showing infective hyphae progressing along *Arachis* testa cells, displaying branching trajectories and transpressoria. (**C**) Sporogenous hyphae advancing both intracellularly and intercellularly, forming early coiling sites. (**D**) Detail of tightly packed sporogenous hyphae growing both intracellularly and intercellularly. (**E**,**F**) SEM images showing sporogenous hyphae developing within host cells and forming compact, rounded early coiled structures. (**G**) SEM details of sporogenous hyphae extending intercellularly between seed-coat parenchyma cells. Symbols: arrowheads indicate hyphae.

As the fungus advanced through the seed coat, infective hyphae initially grew intracellularly within host parenchyma cells (Figure 5A), occupying most of the cytoplasmic space and displacing the host contents. The passage from one cell to the next was facilitated by a transpressorium projection, a narrow protrusion capable of crossing the host cell wall and maintaining continuous intracellular growth (Figure 5B).

These intracellular hyphae subsequently differentiated into sporogenous hyphae, which expanded both within host cells and into the intercellular spaces, forming a continuous network connecting adjacent parenchyma layers (Figure 5C). These hyphal masses grow simultaneously in intracellular and intercellular compartments (Figure 5D–G). Sporogenous hyphae displayed local swellings and incipient coiling, marking the onset of teliosporogenesis (Figure 5C,D).

### 3.6. Intercellular Formation of Coiled Balls

**Figure 6:**
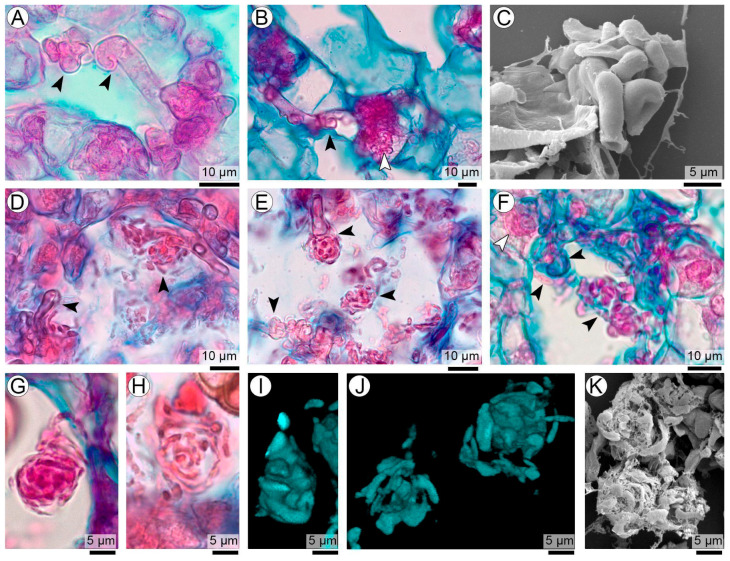
Early formation of intercellular coiled balls during initial teliosporogenesis of *T. frezzii* (**A**,**B**,**D**–**H**: LM). (**A**) Early intercellular coiling of sporogenous hyphae, with thickened hyphal tips beginning to bend and fold within the spaces between seed-coat parenchyma cells. (**B**) Early coiling occurs in both intercellular and intracellular compartments. (**C**) SEM image showing compact early coiled-ball hyphal masses. (**D**–**F**) Intermediate coiling stages with increasing compaction and multilayered turns. (**G**,**H**) Highly compact coiled balls with tightly folded hyphal segments. (**I**,**J**) CLSM Calcofluor images illustrating the three-dimensional arrangement of tightly coiled hyphal masses. (**K**) SEM image of dense aggregates of coiled balls occupying expanded intercellular spaces. Symbols: arrowheads indicate hyphae.

Intercellularly, the process begins with the active proliferation of sporogenous hyphae. These fungal elements advance through the intercellular spaces of the seed-coat parenchyma. As colonization progresses, the apical regions of these hyphae thicken and begin to coil, giving rise to compact fungal aggregates, here termed *coiled balls* (Figure 6A–K). These structures exhibit a broad developmental spectrum, from loosely organized coils to highly compact forms (Figure 6D–K). Newly formed coiled balls are generally less condensed and frequently remain physically connected to the parental sporogenous hyphae by thin, hyaline filaments (Figure 6D–F). This persistent connection indicates an active developmental and nutritional phase prior to the acquisition of the final teliospore morphology and the progressive loss of continuity with the parental sporogenous hyphae (Figure 6D–H).

### 3.7. Intracellular Formation of Coiled Balls and Host-Cell Lysis

**Figure 7:**
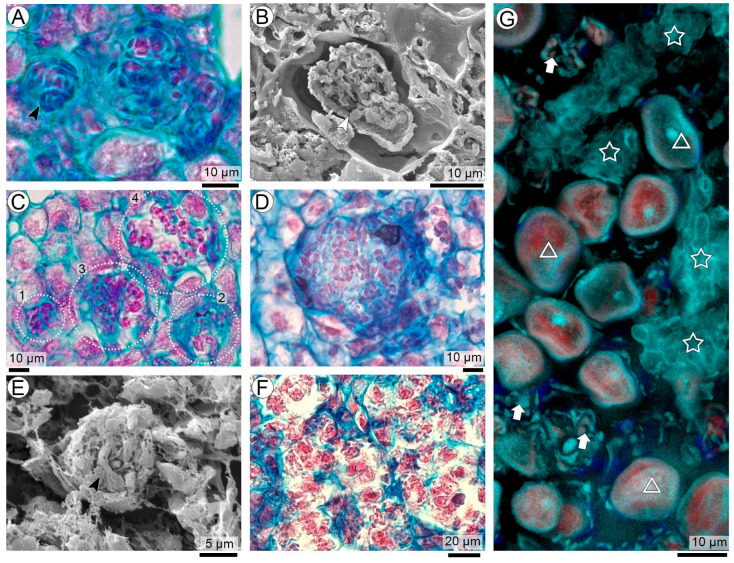
Intracellular formation, maturation, and collapse of coiled balls of *T. frezzii* (**A**,**C**,**D**,**F**: LM). (**A**) Hypertrophied host cell containing a dense Astra Blue–stained mucilaginous matrix with embedded hyphal segments. (**B**) SEM transverse section of an intracellular coiled ball enclosed within the host cell, showing tightly packed hyphal fragments bound by a fibrillar matrix. (**C**) Intracellular sequence (1–4) illustrates progressive cell enlargement, cytoplasmic disintegration, and continued hyphal coiling leading to multiple coiled balls. (**D**) Advanced hypertrophy prior to lysis, with coiled balls immersed in a mucilaginous matrix. (**E**) SEM view of a mature coiled ball released into the extracellular space after host-cell collapse, retaining its compact fibrillar structure. (**F**) Numerous intracellular coiled balls liberated into the collapsed host-cell space. (**G**) CLSM image showing coiled balls (arrows), developing teliospore balls (triangles) embedded within the remnants of lysed seed-coat parenchyma (stars) following host-cell collapse. Symbols: arrowheads indicate hyphae.

Sporogenous hyphae occupying *Arachis* parenchyma cells eventually fill the entire cytoplasmic space, inducing a marked hypertrophy of the invaded host cells (Figure 7A,B). Within these enlarged cells, hyphal masses coil, giving rise to one or several compact intracellular coiled balls (Figure 7C). Their dense organization prevents the visualization of any physical connection with the originating sporogenous hyphae. Simultaneously, this hypha acquires an appearance of multiple closely packed hyphal segments embedded in a matrix that stains intensely with Astra Blue, consistent with a mucilaginous material associated with cellulose-rich walls (Figure 7D). SEM observations are consistent with this observation revealing hyphal fragments of coiled balls held together by a fibrillar matrix, providing ultrastructural evidence of the cohesive material responsible for maintaining the compact architecture of these early teliosporogenic structures (Figure 7E).

Finally, the combination of hyphal compaction, the accumulation of mucilaginous matrix, and host-cell hypertrophy is inferred to generate internal mechanical stress that culminates in host-cell lysis, resulting in the release of fungal material into the extracellular environment (Figure 7F). Following lysis, the mucilaginous matrix gradually dissipates, and only remnants of the disrupted host cell remain. At this point, intracellularly formed coiled balls become morphologically indistinguishable from those formed intercellularly (Figure 7G).

### 3.8. Transition from Coiled Balls to Young Teliospore Balls

**Figure 8:**
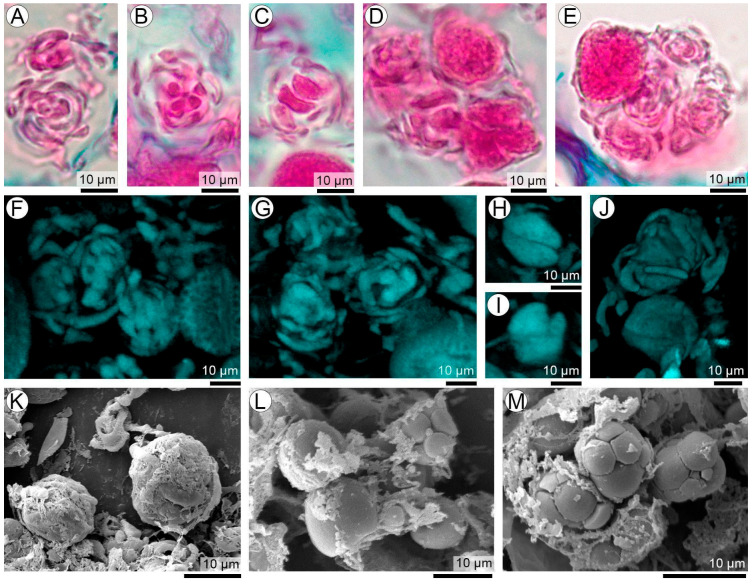
Transition from coiled balls to young teliospore balls of *T. frezzii*. (**A**–**E**) LM images showing progressive compaction and internal differentiation of coiled balls: intercellular coiled balls remain loosely organized and often connected to sporogenous hyphae, whereas intracellular coiled balls are highly compact and lack visible parental connections. (**F**–**J**) CLSM Calcofluor images illustrating the three-dimensional structure of coiled balls at different degrees of compaction and rounding, marking the onset of young teliospore-ball differentiation. (**K**–**M**) SEM images showing coiled balls in advanced stages of rounding, where the central hyphal segments become densely packed and begin to form young teliospore balls, while peripheral hyphae lose cytoplasmic content and become fibrillar.

Regardless of whether coiled balls originate intracellularly or intercellularly, their maturation follows a remarkably consistent developmental pattern that culminates in the formation of young teliospore balls (Figure 8A–M). A clear central–peripheral differentiation becomes evident early in this transition (Figure 8A–C,F–H). The hyphal fragments located at the core of each coiled ball show dense cytoplasm and signs of continued growth; these central elements progressively round up, marking the initial step toward the individualization of future teliospores. In parallel, the peripheral hyphae surrounding the developing center gradually lose their cytoplasmic contents and become increasingly hyaline (Figure 8B–E,G–J). This peripheral layer appears to function as a transient structural scaffold: as the central units mature, the depleted peripheral hyphae collapse or progressively disintegrate, creating the space needed for the expansion of the young teliospores (Figure 8K–M).

As teliosporogenesis progresses, coiled balls and young teliospore balls can be observed at various stages within the host tissue. Fully formed young teliospore balls appear free within the remnants of the hyperplastic seed coat yet remain enclosed by the surrounding epidermal layers, ensuring their retention inside the infected seed (Figure 3B–D and Figure 7G).

### 3.9. Maturation from Young to Fully Developed Teliospores

**Figure 9:**
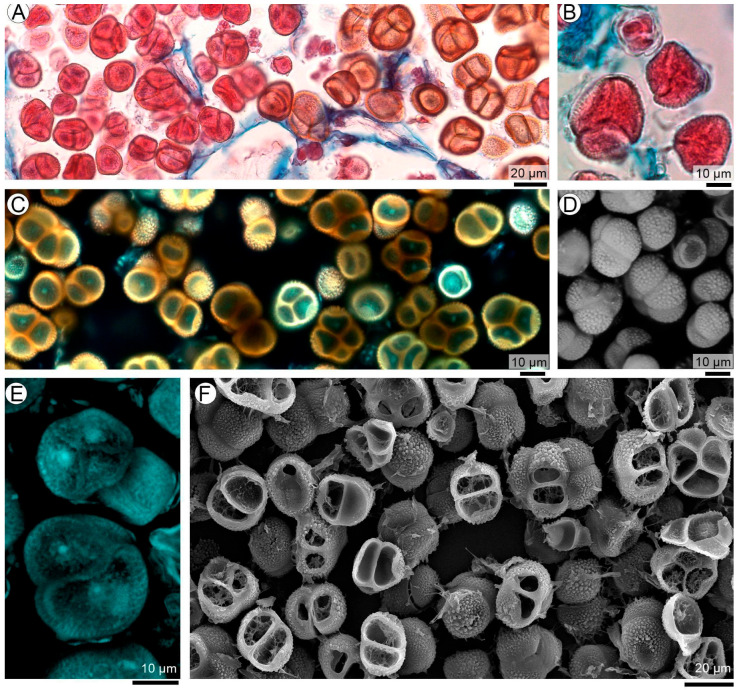
Development and maturation of *T.hecaphora frezzii* teliospore balls. (**A**) Young (left) and mature (right) teliospore balls co-occurring within the same LM field, illustrating the progressive increase in teliospore size accompanied by marked wall thickening and a gradual change in wall coloration during maturation. (**B**) Detail of young teliospores remaining firmly adhered to one another, forming permanent teliospore balls. (**C**) CLSM autofluorescence image showing mature teliospore balls composed of 2–5 cells, each with visible nuclei and pronounced localized wall thickening at the pore region. (**D**) CLSM 3D stack showing teliospore balls composed of 2–5 teliospores. (**E**) CLSM Calcofluor image of young teliospores exhibiting nuclei and early wall differentiation. (**F**) SEM image of mature teliospores showing the fully developed echinulate wall pattern.

Despite the variability in their origin—whether coiled balls arise intercellularly or within the cytoplasm of host parenchyma cells—and regardless of the embryogenic stage at which infection occurs (R4–R5 in young seeds, or R6–R8 in maturing ones), teliosporogenesis follows a conserved developmental pattern (Figure 8A–J). Within each precursor coiled ball, a defined number of central hyphal units (typically two to seven) enlarge, round up, and differentiate into young teliospores. The resulting teliospores remain closely associated, retained by remnants of the intervening matrix, forming the definitive teliospore balls (Figure 9A–F). Each teliospore ball generally contains a small number of spores, most commonly two to four, with a diameter of 21.47–30.29 μm (SD: 3.09; $\bar{X}$ = 25.89 μm). Less frequently, teliospore balls may contain up to seven spores, reaching a maximum diameter of 37.07–37.97 μm (SD: 0.64; $\bar{X}$ = 37.53 μm).

Mature teliospores show distinctive wall features. Their walls are markedly thickened, likely conferring increased mechanical resistance, with an echinulate ornamentation on the exposed, rounded surfaces and smooth, straight walls at the spore–spore contact zones (Figure 9A,C,D,F). In LM with safranin–Astra Blue staining, immature teliospores appear light pink to reddish, while mature teliospores show a more intense, orange-brown staining, consistent with progressive wall thickening (Figure 9A,B). As the number of teliospores per ball increases, and as teliospore balls accumulate within the hypertrophied seed coat, the parenchyma is progressively replaced by densely compacted masses of mature teliospores (Figure 2C and Figure 3D).

## 4. Discussion

### 4.1. Correlative and Multiscale Microscopy in the Peanut Smut Pathosystem

Correlative light and electron microscopy (CLEM) has undergone extensive development and has become a key strategy in cell biology, particularly for resolving subcellular structures at the ultrastructural level or under highly controlled experimental conditions [27,28,29,30]. Its main—and perhaps most limiting—feature is its technical complexity, as it typically requires cryo-fixation or freeze substitution protocols, fluorescence-compatible embedding media, spatial reference markers for image alignment, and high-end integrated imaging platforms [26,45,46]. Moreover, recent multiscale adaptations that incorporate stereomicroscopy as a bridge to anatomical context continue to rely on specialized chambers, integrated systems, or custom-designed reagents [47,48,49]. As a consequence, the broader application of CLEM remains largely restricted to highly equipped laboratories, limiting its routine use in structurally complex biological systems.

In contrast, the workflow developed here constitutes a pragmatic correlative multiscale strategy based on broadly accessible histological and microscopy techniques. By applying stereomicroscopy, light microscopy (LM), confocal laser scanning microscopy (CLSM), and scanning electron microscopy (SEM) sequentially to serial sections obtained from a single paraffin-embedded tissue block, this approach preserves anatomical continuity and enables spatial correlation at the level of tissues and biological structures, rather than at the level of an identical microscopic field. This design follows the conceptual framework proposed by Caplan et al. [27], in which correlation is achieved through the integration of defined biological targets, anatomical context, complementary contrast mechanisms, and increasing spatial resolution, without requiring exact field coincidence across modalities. The workflow builds on classical histological methods [32,33,37], which have been successfully applied to complex plant–fungus systems, including the detection and tissue-level localization of a fungal endophyte in *Astragalus* [50]. In addition, a multiscale variant of this approach was previously used to characterize the progressive colonization of peanut tissues by *T. frezzii* [15], providing the technical foundation that is expanded here into a fully integrated correlative design.

Crucially, the correlative value of this workflow lies in the non-redundant information gained from the integration of techniques, as illustrated in Figure 1. Light microscopy provides the histological and developmental framework necessary to identify fungal structures in situ and to relate them to host tissue organization. However, LM alone is inherently limited by optical depth and two-dimensional sectioning. SEM observations performed directly on histological sections extend the depth of field and reveal the true three-dimensional architecture of fungal aggregates within host tissues, clarifying whether observed profiles correspond to isolated sections or to compact, spatially coherent structures. In addition, SEM resolves surface topography and structural details that are not evident in transmitted-light LM observations. CLSM further complements these observations by enabling optical sectioning and three-dimensional reconstructions, allowing internal organization, spatial continuity, and volumetric relationships to be resolved. The integration of these datasets yields structural information that cannot be inferred from any single technique in isolation.

Applied to the peanut–*Thecaphora frezzii* pathosystem, this correlative framework enabled the in situ reconstruction of fungal organization across scales, from hyphal networks embedded in host tissues to the spatial architecture of spore balls and mature teliospore balls. Importantly, this integration made it possible to interpret developmental sequences within their anatomical context, demonstrating that detailed multiscale analyses of fungal development can be achieved using widely available histological and microscopy resources. In this sense, the workflow does not merely combine techniques, but exploits their complementarity to extract biologically meaningful information that emerges only through correlation.

### 4.2. Overview of Teliosporogenesis

Teliosporogenesis represents the terminal developmental event of the parasitic phase of *Thecaphora frezzii* and constitutes the transition from biotrophic growth to the production of resistance and dispersal structures. In infected peanuts, this process is macroscopically manifested by the replacement of the seeds with a dark brown, powdery teliospore mass.

**Figure 10:**
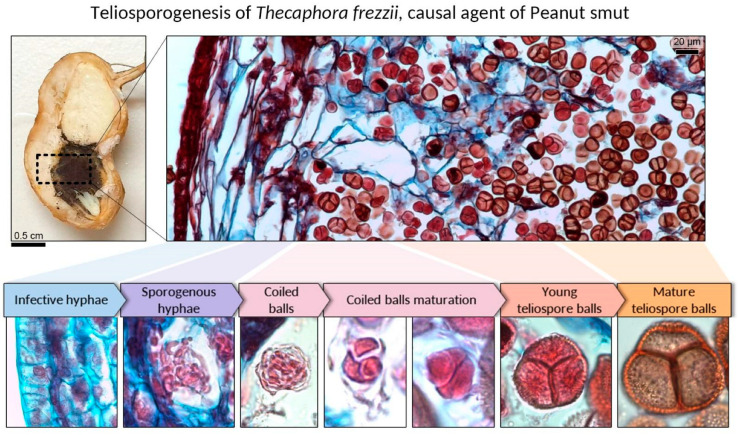
Teliosporogenesis of *Thecaphora frezzii* in peanuts. Longitudinal section of an infected fruit highlighting the seed coat as the site of sporulation; the boxed region is enlarged to show the hyperplastic testa containing abundant teliospore balls. Below, representative insets summarize the successive developmental stages documented in this study: infective hyphae, sporogenous hyphae, formation of coiled balls, progressive compaction/maturation of coiled-ball precursors, young teliospore balls, and mature teliospore balls. Insets are aligned with the corresponding tissue zone to emphasize the spatial continuity of the process within the seed coat.

Figure 10 summarizes the main morphogenetic transitions observed in situ, providing an overview of the shift from vegetative growth to the sporogenic phase. The sequence begins with the differentiation of the *infective hyphae* into *sporogenous hyphae* within the hyperplastic and hypertrophic testa. Sporogenous hyphae then reorganize, undergo progressive compaction, and segment and coil, forming three-dimensional coiled balls in both intercellular spaces and within parenchyma cells. As maturation proceeds, hyphae fragments and the individual cells at the center of the coil begin to round off and enlarge as teliospore initials, while peripheral hyphae become hyaline and collapse. As host tissues lysis progress, the maturation of the *young teliospores* is completed, reaching their definitive state as thick-walled, ornamented teliospores. Final maturation is characterized by pronounced wall thickening and the development of the typical ornamented teliospores that remain permanently aggregated as *mature teliospore balls*.

### 4.3. Teliosporogenesis in Thecaphora frezzii: Host Dependence and Ontogenetic Progression

The dependence of *Thecaphora frezzii* on the developmental stage of the host is consistent with its specialized biotrophic lifestyle and places it among smut fungi that require precise synchronization with host phenological stages [51,52]. This dependence was clearly addressed in *Ustilago maydis*, in which teliosporogenesis is activated exclusively in highly differentiated tumorous tissues, as in the leaves, but does not occur in axenic culture [52,53]. Similarly, in *T. frezzii*, the transition from infective hyphae to sporogenous hyphae is observed only once the seed coat has been reached, underscoring the tight coupling between host tissue differentiation and fungal developmental fate.

From a comparative perspective, the functional convergence of the seed as the site of teliosporogenesis emerges as a recurrent trait across different lineages of Ustilaginomycotina, even when the initially colonized organs and infection pathways differ substantially. In systemic smuts such as *Sporisorium reilianum* (J.G. Kühn) Langdon & Full., *Thecaphora thlaspeos* (Beck) Vánky and *U. maydis*, vascular colonization ultimately culminates in the spike or grain, where teliospores partially or completely replace the seed tissues [11,54,55,56,57]. In other cases, such as *Thecaphora deformans*, sporulation may occur in both anthers and seeds, but it is invariably associated with reproductive tissues at advanced developmental stages [24]. Beyond reproductive structures, a similar pattern is observed in species such as *Thecaphora solani* (Thirum. & M.J. O’Brien) Mordue, which induces smut formation in potato stem tubers—vegetative organs involved in asexual reproduction and storage—yet likewise characterized by high resource allocation and metabolic investment [58]. Taken together, these examples suggest that the preference for specific host organs as sites of sporulation reflects a fungal strategy targeting tissues with maximal nutrient availability and developmental commitment, whether reproductive or storage-related, thereby enhancing the efficiency of teliospore production rather than representing a passive outcome of systemic colonization.

Within this framework, our results indicate that teliosporogenesis in *T. frezzii* involves a highly organized sequence of morphogenetic events, associated with a progressive reorganization of hyphal growth. Notably, during the sporulation phase, the observation of intercellular hyphal growth aside the intracellular one revealed a plastic pattern not recorded during earlier stages of the biotrophic cycle [15]. This plasticity reflects an active adjustment of hyphal growth mode to local tissue architecture. In regions of compact seed-coat parenchyma, hyphae grow predominantly intracellularly, whereas in areas of the same tissue with well-developed intercellular spaces, growth shifts to an intercellular pattern. The intracellular continuity is facilitated by the formation of transpressoria, specialized hyphal projections that enable direct cell-to-cell passage across host walls, as previously documented for *T. frezzii* [15]. This hyphal growth plasticity has only rarely been documented in Ustilaginomycetes and has never been reported in *Thecaphora*. Previous studies on other species of this genus have focused mainly on the general localization of infection—such as flowers (ovaries, anthers), meristematic shoots, or tubers—without detailing the fine-scale patterns of hyphal growth during sporogenesis [19,20,21,24,58]. Therefore, the description of a dual intracellular/intercellular growth pattern in *T. frezzii* represents a novel contribution to the understanding of its life cycle and may be crucial for enabling the fungus to accommodate the structural changes accompanying seed development, thereby ensuring efficient colonization until the completion of teliosporogenesis, as reported for other smut fungi [11,22,59,60]

The onset of teliosporogenesis in *T. frezzii* represents a morphogenetic turning point in the pathogen’s life cycle, in which vegetative growth is progressively replaced by a highly organized differentiation program [51,52]. In Ustilaginomycetes, this process marks the transition from the filamentous dikaryotic parasitic phase to the resistant and dispersive phase, culminating in the formation of thick-walled, pigmented teliospores [11,16,17,53]. In *T. frezzii*, this transition does not occur through simple hyphal fragmentation, as described for *Ustilago*, *Microbotryum*, *Tilletia*, and *Entorrhiza*, among other genera that produce solitary teliospores [16,53], but is rather mediated by a prior spatial reorganization of the hyphal system. A distinctive feature of *T. frezzii* is the formation of so-called “coiled balls” reported earlier for other fungi, that consist of highly organized three-dimensional structures composed of densely coiled sporogenous hyphae embedded in a mucilaginous matrix [16,21,22,58,61,62]. These structures represent the earliest stage of hyphal reorganization leading to the formation of permanent teliospore balls at the end of teliosporogenesis in *T. frezzii.* This finding constitutes a significant advance in the understanding of *T. frezzii* development, as it reveals the morphological differentiation through which the biotrophic mycelium ceases invasive growth and reorganizes into a compact three-dimensional structure, thereby initiating the reproductive phase.

Piepenbring et al. [17] provided comparative descriptions of the ultrastructure of mature teliospores and the final organization of teliospore balls in Ustilaginomycetes—including *Thecaphora* Fingerh. and *Sorosporium* —but their analyses were restricted to terminal stages and did not address the ontogenetic events leading to teliospore formation. In the present study, teliospore maturation in *Thecaphora frezzii* is resolved in situ by following the progressive differentiation of the teliospore wall. When stained with Safranin–Astra Blue, immature teliospores show a weak to moderate affinity for safranin, whereas mature teliospores exhibit a markedly stronger and darker staining response. Although this chromatic progression is induced by the staining reaction and does not constitute direct chemical evidence of pigment deposition, it is consistent with the wall hardening and dark pigmentation classically associated with smut teliospores and commonly interpreted as melanization or melanization-like modification of the teliospore wall, without implying direct chemical identification of melanin [52,63,64]. At the macroscopic level, this dark pigmentation is responsible for the characteristic carbonaceous appearance of peanut grains, affected by smut, a feature long documented in pathological and agronomic studies of the disease [11,12,65].

### 4.4. Comparative Framework and the Teliospore-Ball Type

Classical characters traditionally used in smut taxonomy—such as the organ in which the sorus develops, the presence or absence of peridium and columella, the arrangement of teliospores (solitary, paired, or aggregated in balls), host specificity, teliospore morphology, and the color of the carbonaceous mass—exhibit high variability, which has limited their systematic utility mainly to generic or higher taxonomic levels delimitations [19]. The final architecture and wall features of mature teliospores in *Thecaphora* have already been described in detail by Piepenbring and collaborators [17], and our observations fully corroborate those accounts; therefore, the present discussion deliberately shifts the focus from terminal spore morphology to the ontogenetic processes underlying teliospore-ball formation.

The ontogenetic schemes proposed by Piepenbring [16] recognize four classical types of teliosporogenesis associated with the formation of solitary teliospores (*Ustilago*, *Microbotryum*, *Tilletia*, and *Entorrhiza* types), which are primarily defined by patterns of hyphal segmentation and by the presence or absence of a hyaline matrix. Although spore-ball-forming genera such as *Thecaphora* and *Sporisorium* Ehrenb. ex Link, are mentioned within the *Ustilago* type, the ontogenetic mechanisms leading to spore-ball formation are not explicitly addressed, and the developmental pattern of these aggregated structures remains unresolved. The developmental sequence observed here in *T. frezzii*, in which teliosporogenesis is preceded by an organized phase of hyphal coiling and the formation of well-defined precursor structures, does not fit any of the four types described by Piepenbring [16] for smut fungi.

Taken together, the evidence indicates that the “coiled balls” observed in *T. frezzii* function as key precursor structures, ensuring an ordered spatial distribution of teliospores and a largely synchronous differentiation process. The pattern documented here in *T. frezzii* shows a clear affinity with the model described by Langdon and Fullerton [22] for *Sorosporium*, based on the early formation of “sporogenous hyphal coils” that act as progenitor units of teliospore balls. In that model, the active spatial organization of sporogenous hyphae into coils, followed by wall gelatinization and loss of septal identity, governs the formation of permanent teliospore balls, which represent developmentally programmed structures rather than the product of passive hyphal fragmentation, as in the *Ustilago* type. Although this ontogenetic pattern was not formalized as a distinct category by Piepenbring [16], it closely matches the sequence documented here for *T. frezzii*.

Based on the combined evidence of the present report and observations made by Langdon and Fullerton [22], we propose here a new ontogenetic pattern of teliosporogenesis, designated the Teliospore-ball type, applicable to taxa of Ustilaginomycetes in which teliospores are produced as aggregated balls as the final outcome of the sporogenic process. This ontogenetic type has to be added to those described by Piepenbring [16] and is characterized by:

(i) an organized developmental pathway mediated by an early phase of “coiled balls” that function as precursor units;

(ii) the presence of a mucilaginous matrix associated with progressive hyphal reorganization; and

(iii) subsequent coordinated reorganization and differentiation of these precursor hyphae, with hyphal segmentation occurring as part of the developmental process, giving rise to permanent teliospore balls.

This ontogenetic pattern reflects a higher level of developmental organization than simple linear hyphal segmentation and provides a coherent framework that integrates the morphological, histopathological, and comparative evidence available for smut fungi forming teliospore balls. Despite the ontogenetic similarity, an important structural difference must be noted between *Thecaphora* and *Sorosporium*. In *Sorosporium*, teliospore balls develop within well-defined sori that include soral structures such as a peridium and columella, whereas in *Thecaphora* species teliospore balls form in sori lacking these structures [19,20,21,66]. Thus, the present study provides the first detailed ontogenetic description of teliospore-ball formation occurring in the absence of a structured sorus, expanding the known morphological and developmental spectrum of this strategy. Comparative analyses of teliosporogenesis in additional genera producing aggregated teliospores will be necessary to evaluate the scope and general applicability of the Teliospore-ball type within Ustilaginomycetes that form teliospore balls.

## 5. Conclusions

This study demonstrates that a correlative and multiscale microscopy workflow based on routine, widely accessible histological techniques can effectively integrate structural information across scales in the peanut–*Thecaphora frezzii* pathosystem. By applying stereomicroscopy, light microscopy, confocal laser scanning microscopy, and scanning electron microscopy sequentially to serial sections from a single paraffin block, coherent spatial correlation was achieved from organ-level anatomy to fine structural detail, without reliance on specialized or integrated instrumentation. This approach provides a robust and scalable methodological framework for the in situ analysis of fungal structures within host tissues and can be readily applied to the study of morphogenetic and pathological processes in structurally complex host–pathogen systems.

Beyond its methodological contribution, the results establish a comprehensive ontogenetic framework for teliosporogenesis in *Thecaphora frezzii*, grounded in in situ multiscale evidence. Teliospore formation was shown to be a highly regulated developmental process, strictly dependent on host tissue context and developmental stage, rather than a passive outcome of hyphal fragmentation. The transition from biotrophic growth to sporulation involves an early and organized reprogramming of hyphal architecture, marked by the formation of three-dimensional “coiled balls” embedded in a mucilaginous matrix, which function as precursor structures and ultimately give rise to permanent teliospore balls as the final outcome of the sporogenic process.

Placed within a comparative perspective, these findings highlight the limitations of classical ontogenetic schemes of teliosporogenesis based solely on terminal spore morphology. The developmental sequence documented here supports recognition of a distinct ontogenetic pattern—the Teliospore-ball type—characterized by early hyphal coiling, matrix-mediated reorganization, and a coordinated reorganization of sporogenous hyphae culminating in the formation of permanent teliospore balls. By integrating multiscale imaging with ontogenetic interpretation, this study contributes both a transferable methodological strategy and a refined conceptual model for understanding developmental diversity in Ustilaginomycetes. Comparative evidence from other smut genera suggests that this ontogenetic pattern may extend beyond *Thecaphora*, underscoring its potential relevance within Ustilaginomycetes.

## Data Availability

The original contributions presented in this study are included in the article.
